# Supplementary material for: Empirical methods for controlling false positives and estimating confidence in ChIP-Seq peaks
Source: BMC Bioinformatics. 2008 Dec 5;9:523. doi: 10.1186/1471-2105-9-523 (PMC2628906; doi:10.1186/1471-2105-9-523)
Supplement: Additional File 1 — A variety of html documents from the USeq web site detailing the available applications, their best usage, output file type descriptions, command line menus, etc. [file 1471-2105-9-523-S1.zip › USeqUserGuides/JavaDoc.zip!/JavaDoc/expr/CorrelationWindow.html]

CorrelationWindow


|  |  |  |
| --- | --- | --- |
| **Overview** \| **Package** \| **Class** \| **Use** \| **Tree** \| **Deprecated** \| **Index** \| **Help** | |  |
| **PREV CLASS**   **NEXT CLASS** | **FRAMES**    **NO FRAMES**     **All Classes** |  |
| SUMMARY: NESTED \| FIELD \| CONSTR \| METHOD | DETAIL: FIELD \| CONSTR \| METHOD |  |


---


## expr Class CorrelationWindow

```
java.lang.Object
  expr.CorrelationWindow
```

---

``` public class CorrelationWindow extends java.lang.Object ```

---

**Constructor Summary**

`CorrelationWindow(float score,
java.lang.String chromosome,
int start,
int stop,
java.lang.String[] names)`


| **Method Summary** | |
| --- | --- |
| `java.lang.String` | `getChromosome()` |
| `java.lang.String[]` | `getNames()` |
| `float` | `getPvalue()` |
| `float` | `getScore()` |
| `int` | `getStart()` |
| `int` | `getStop()` |
| `void` | `setPvalue(float pvalue)` |
| `java.lang.String` | `toString()`             Genes NumGenes Chromosome Start Stop CorrelationScore -10Log10(pvalue) |

**Methods inherited from class java.lang.Object**

`equals, getClass, hashCode, notify, notifyAll, wait, wait, wait`

**Constructor Detail**

### CorrelationWindow

```
public CorrelationWindow(float score,
                         java.lang.String chromosome,
                         int start,
                         int stop,
                         java.lang.String[] names)
```


**Method Detail**

### toString

```
public java.lang.String toString()
```

:   Genes NumGenes Chromosome Start Stop CorrelationScore -10Log10(pvalue)

    :   **Overrides:**: `toString` in class `java.lang.Object`

---


### getPvalue

```
public float getPvalue()
```

---


### setPvalue

```
public void setPvalue(float pvalue)
```

---


### getNames

```
public java.lang.String[] getNames()
```

---


### getScore

```
public float getScore()
```

---


### getStart

```
public int getStart()
```

---


### getStop

```
public int getStop()
```

---


### getChromosome

```
public java.lang.String getChromosome()
```


---


|  |  |  |
| --- | --- | --- |
| **Overview** \| **Package** \| **Class** \| **Use** \| **Tree** \| **Deprecated** \| **Index** \| **Help** | |  |
| **PREV CLASS**   **NEXT CLASS** | **FRAMES**    **NO FRAMES**     **All Classes** |  |
| SUMMARY: NESTED \| FIELD \| CONSTR \| METHOD | DETAIL: FIELD \| CONSTR \| METHOD |  |


---
